# Development of an all-in-one real-time PCR assay for simultaneous detection of spotted fever group rickettsiae, severe fever with thrombocytopenia syndrome virus and hantaan virus prevalent in central China

**DOI:** 10.1371/journal.pntd.0012024

**Published:** 2024-07-16

**Authors:** Cuixiang Wang, Liangjun Chen, Xingrong Li, Jihong Gu, Yating Xiang, Liang Fang, Lili Chen, Yirong Li

**Affiliations:** 1 Department of Laboratory Medicine, Zhongnan Hospital of Wuhan University, Wuhan University, Wuhan, China; 2 Department of Wuhan EasyDiagnosis Biomedicine, Wuhan, China; 3 Wuhan Research Center for Infectious Diseases and Cancer, Chinese Academy of Medical Sciences, Wuhan, People’s Republic of China; 4 Hubei Engineering Center for Infectious Disease Prevention, Control and Treatment, Wuhan, People’s Republic of China; Foshan University, CHINA

## Abstract

Central China has been reported to be one of the most important endemic areas of zoonotic infection by spotted fever group rickettsiae (SFGR), severe fever with thrombocytopenia syndrome virus (SFTSV) and hantaan virus (HTNV). Due to similar clinical symptoms, it is challenging to make a definite diagnosis rapidly and accurately in the absence of microbiological tests. In the present study, an all-in-one real-time PCR assay was developed for the simultaneous detection of nucleic acids from SFGR, SFTSV and HTNV. Three linear standard curves for determining SFGR-*ompA*, SFTSV-L and HTNV-L were obtained within the range of 10^1^−10^6^ copies/μL, with the PCR amplification efficiencies ranging from 93.46% to 96.88% and the regression coefficients R^2^ of >0.99. The detection limit was 1.108 copies/μL for SFGR-*ompA*, 1.075 copies/μL for SFTSV-L and 1.006 copies/μL for HTNV-L, respectively. Both the within-run and within-laboratory coefficients of variation on the cycle threshold (Ct) values were within the range of 0.53%-2.15%. It was also found there was no statistical difference in the Ct values between single template and multiple templates (P_**SFGR-*ompA***_ = 0.186, P_SFTSV-L_ = 0.612, P_HTNV-L_ = 0.298). The sensitivity, specificity, positive and negative predictive value were all 100% for determining SFGR-*ompA* and SFTSV-L, 97%, 100%, 100% and 99.6% for HTNV-L, respectively. Therefore, the all-in-one real-time PCR assay appears to be a reliable, sensitive, rapid, high-throughput and low cost-effective method to diagnose the zoonotic infection by SFGR, SFTSV and HTNV.

## Author summary

Spotted fever (SF), severe fever with thrombocytopenia syndrome (SFTS), and hemorrhagic fever with renal syndrome (HFRS) sporadically have outbreaks in central China. Due to the similarities in clinical symptoms and the absence of reliable diagnostic methods for the detection these pathogens simultaneously, clinical diagnosis and treatment frequently result in misdiagnosis or missed diagnosis. Thus, the development of a fast and accurate diagnostic method is crucial for prevention and precise treatment. In this study, we designed an all-in-one real-time PCR assay to differentiate spotted fever group rickettsiae (SFGR), severe fever with thrombocytopenia syndrome virus (SFTSV) and hantaan virus (HTNV). The *ompA* gene of SFGR, as well as the segment L of SFTSV and HTNV, were used as targets to design primers and probes for amplification. Through the verification of nucleic acid and clinical sample detection, the sensitivity of this detection method exceeded 97%, and its specificity was 100%.

This new assay could be applied in epidemiology and clinical diagnosis, to control new outbreaks, reduce diagnostic and identification time, and improve test efficiency.

## Introduction

Zoonotic diseases typically occur sporadically and are more prevalent in economically underdeveloped areas, such as remote mountainous and forested regions [1–3]. Due to the limited medical laboratory resources and similar clinical symptoms, timely and accurate diagnosis of zoonotic diseases is often difficult and it leads to inadequate and untimely treatment. Since 2009, it has been found that there is a high prevalence of severe fever with thrombocytopenia syndrome (SFTS) in central China including Dabie and Yiling Mountains region [4,5]. SFTS is a zoonotic disease caused by a tick-borne virus called severe fever with thrombocytopenia syndrome virus (SFTSV), a novel *Bandavirus* of family *Phenuiviridae*, which was recently named *Dabie Bandavirus* by the International Committee on Taxonomy of Viruses (ICTV). The main clinical manifestations include acute fever, thrombocytopenia, leukopenia, gastrointestinal and neurological symptoms [6–8]. Moreover, multiple organ failure may occur in severe cases with a maximum mortality of 30% [9,10]. Recently, spotted fever (SF), another zoonotic infectious disease caused by the spotted fever group rickettsiae (SFGR), was found in succession in central China. SFGR is an intracellular bacteria belonging to the spotted fever group (SFG) of the genus *Rickettsia* in the family *Rickettsiaceae* [11]. It was reported that the seroprevalence rate of anti-*Rickettsia japonica* antibody is about 21% among people in Yichang, a city in the Yiling Mountains region of central China [12]. The main clinical symptoms of tick-borne rickettsioses also include fever and thrombocytopenia as well as headache, muscle pain, rash and local lymphadenopathy [12–17]. It is worth noting that central China including the Dabie and Yiling Mountains region is also known to be an important endemic area for hantaan virus (HTNV) infections transmitted by rodents, which was recently named orthohantavirus hantanense by ICTV belonging to the order *Bunyavirales*, family *Hantaviridae*, and genus *Orthohantavirus* [18]. The main epidemic strain of HTNV is HV004 in the past ten years [18–20]. Hemorrhagic fever with renal syndrome (HFRS), caused by HTNV, is characterized by a combination of symptoms, which include fever, hemorrhage, thrombocytopenia and acute kidney injury [21,22]. The early clinical manifestations of SF, SFTS and HFRS are often similar and nonspecific, with most patients experiencing systemic symptoms such as fever, thrombocytopenia, headache, fatigue and muscle aches [23,24]. Therefore, it is challenging to rapidly and accurately identify these pathogens in febrile patients with thrombocytopenia and a history of outdoor activities in central China based on the clinical symptoms.

There are a variety of methods including antigen-antibody detection and nucleic acid testing to be used for the identification of microbial pathogens. Antigen-antibody detection such as Enzyme-linked immunosorbent assay and indirect immunofluorescence are limited in the precise diagnosis due to wide antigen cross-reactivity and delayed seroconversion [25–27]. Nucleic acid testing has always been considered to be the preferred method for diagnosing viral infections, including quantitative real-time fluorescence PCR assay, isothermal amplification reaction, digital PCR assay and **metagenomics next generation sequencing** (mNGS). Although isothermal amplification reactions is rapid and do not require a specialized thermocycler, it have high requirements for primer designing and serious challenges for a multiplex PCR assay. Digital PCR assay and mNGS have their advantages in absolute quantification and unbiased microbial infection, respectively, but it is not unsuitable to carry out in hospitals located in economically underdeveloped remote areas due to their high cost or time-consuming procedure. It has been reported that the real-time PCR assay is the preferred choice for detecting the nucleic acid of viruses due to its high accuracy, faster testing time and lower cost. It was also found that there was only one commercially available kit for testing target nucleic acids of SFTSV. Therefore, in order to quickly diagnose these zoonotic diseases caused by SFGR, SFTSV and HTNV in central China, we established a rapid, convenient and accurate all-in-one real-time PCR assay following the evaluation of clinical performances.

## 2. Materials and methods

### 2.1 Ethics statement

All samples were collected as part of public health diagnostic activities for SF, SFTS,and HFRS were pre-existing relative to the start of the study, and were examined as anonymous samples. In accordance with the medical research regulations in China, the research scheme was authorized by the medical Ethics Committee of Zhongnan Hospital of Wuhan University (No. 2023052K).

### 2.2 Serum or nucleic acid samples

A total of 321 serum or nucleic acid samples were collected in the present study. Of them, 17 SFGR DNA-positive nucleic acid samples were obtained from Beijing Center for Disease Control and Prevention (CDC) (n = 9) and State Key Laboratory of Virology (n = 8), 33 HTNV RNA-positive nucleic acid samples were prepared in State Key Laboratory of Virology (n = 21) and Hubei CDC (n = 12), the remaining including 46 SFTSV RNA-positive nucleic acid samples and 225 serum samples without three target pathogens were garnered from Zhongnan Hospital of Wuhan University. These samples were previously tested to be positive for SFTSV, hepatitis B virus (HBV), hepatitis C virus (HCV), Epstein-Barr virus (EBV) and cytomegalovirus (CMV) by commercially available kits (DaAn Gene Co., Ltd), for SFGR DNA and HTNV RNA by Sanger sequencing after nested PCR, respectively [12,18]. The study was approved by the Ethics Committee of Zhongnan Hospital of Wuhan University.

### 2.3 Primers and probes

**Fig 1:**
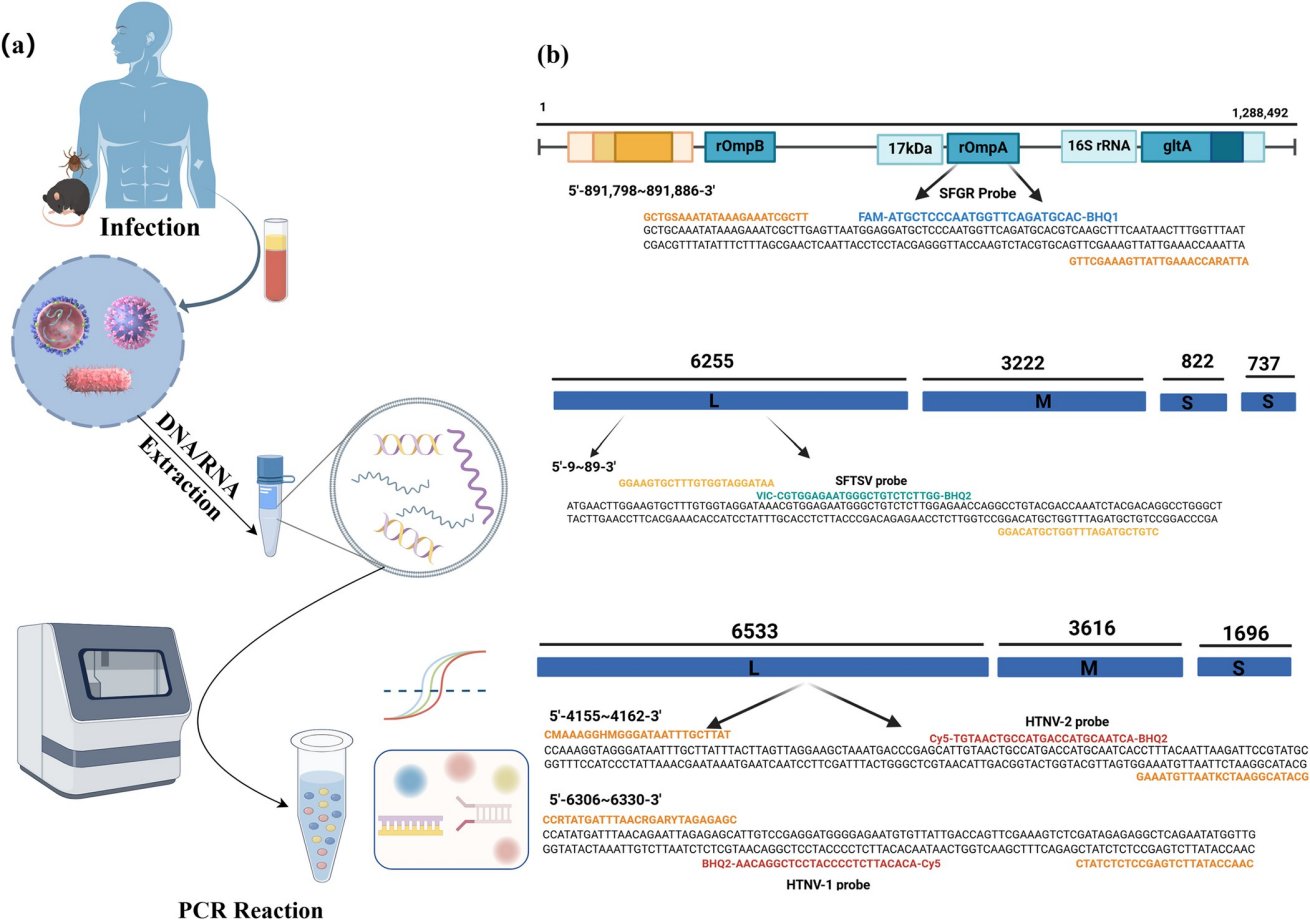
Schematic illustration of the proposed all-in-one real-time PCR assay. (a) Detection principle schematic diagram; (b) Schematic of the SFGR, SFTSV and HTNV genome and corresponding primer sequence design. (Created by Figdraw., export ID:UTYWAc96d8).

All primers and probes were synthesized by General Biosystems (Anhui, China). The detailed primers and probes sequences are listed in **Table 1**. The primers ompA-F/R, and the probe ompA-P were used to amplify the target *ompA* gene in the SFGR (including *R*. *japonica*, *R*. *raoultii*, *R*. *heilongjiangensis*, *R*. *conorii*, *R*. *sibirica*, *R*. *slovaco*, *Candidatus Rickettsia* and so on). The 5′-ends and 3′-ends of ompA-P were labeled with FAM (6-carboxyfluorescein) and **Black Hole Quencher 1** (BHQ1**)**, respectively. The primer pair DL-F/R, and the probe DL-P were employed to amplify the segment L in SFTSV. The VIC (5-VIC phosphoramidite) and **Black Hole Quencher 2** (BHQ2) were labeled in the 5′-ends and 3′-ends of DL-P, respectively. Two sets of primers and probes including HL-F1/R1 and HL-P1, and HL-F2/R2 and HL-P2, were simultaneously adopted to amplify the segment L in HTNV, and both the 5′-ends and 3′-ends of HL-P1 as well as HL-P2 were labeled with Cy5 (Cyanine 5) and BHQ2, respectively. *Beta-actin* (*ACTB*) was used as the endogenous reference gene in the all-in-one real-time PCR assay. ACTB-F1, ACTB-R1 and ACTB-P were employed to amplify *ACTB*, and the 5′-ends and 3′-ends of ACTB-P were labeled with ROX (5-Carboxy-X-rhodamine) and BHQ2. All of the primers and probes for amplifying *ompA* and L were designed using Primer Premier 5.0 design software, whereas those for amplifying the *ACTB* were the same as those used in previous studies (**Fig 1**) [28].

**Table 1:** Primers and probes used in the all-in-one real-time PCR assay.

pathogen	Primers and probes	Nucleotide sequence (5′-3′)	Gene or segments	Position	Product length (bp)
**SFGR**	ompA-probe	FAM-ATGCTCCCAATGGTTCAGATGCAC-BHQ1	*ompA*	1176385-1176473^a^	89
	ompA-F	GCTGSAAATATAAAGAAATCGCTT			
	ompA-R	ATTARACCAAAGTTATTGAAAGCTTG			
**SFTSV**	DL-probe	VIC-CGTGGAGAATGGGCTGTCTCTTGG-BHQ2	L	9-89^b^	81
	DL-F	GGAAGTGCTTTGTGGTAGGATAA			
	DL-R	CTRTCGTAGATTTGGTCRTACAGG			
**HTNV**	HL-probe1	Cy5-ACACATTCTCCCCATCCTCGGACAA-BHQ2	L	6278-6377^c^	100
	HL-F1	CCRTATGATTTAACRGARYTAGAGAGC			
	HL-R1	CAACCATAYTYTGAGCCTCTCTATC			
	HL-probe2	Cy5-TGTAACTGCCATGACCATGCAATCA-BHQ2		4155-4262^d^	108
	HL-F2	CMAAAGGHMGGGATAATTTGCTTAT			
	HL-R2	GCATACGGAATCKTAATTGTAAAG			
	ACTB-probe	ROX-ACCACCACGGCCGAGCGG-BHQ2		648-706^e^	59
**ACTB**	ACTB-F	GAGCGCGGCTACAGCTT	*ACTB*		
	ACTB-R	TCCTTAATGTCACGCACGATTT			

a:AE006914.1; b:NC_043450.1; c:NC_005222.1; d:JQ083393.1; e:KR710455.1.

### 2.4 DNA/RNA extraction

DNA and RNA were extracted according to the instructions of the Vazyme Fast Pure Viral DNA/RNA Mini Kit Pro (Vazyme Biotech Co.,Ltd). All of the nucleic acid samples used in the present study were stored at -80°C until further experiments.

### 2.5 Construction of the plasmids

Plasmids SFGR-*ompA*, SFTSV-L, HTNV-L1 and HTNV-L2 constructed by General Biol (Anhui) Co., Ltd were used to create standard curves and determine the limit of detection (LOD) for the all-in-one real-time PCR assay. The conserved regions sequences used for constructing the plasmids are seen in **S1 Table**. The copy number of plasmid was calculated using the formula: plasmid copy number (copies/μL) = plasmid concentration × 10^−9^ × diluted multiples × 6.02 × 10^23^) / (660 Dalton/bases × DNA length in nucleotides). The initial concentrations of these plasmids were determined to be 6.92 × 10^9^ copies/μL for SFGR-*ompA*, 5.30 × 10^9^ copies/μL for SFTSV-L, 6.80 × 10^9^ for HTNV-L1 and 5.79 × 10^9^ copies/μL for HTNV-L2, respectively. All of them were stored at -80°C for future experiments.

### 2.6 All-in-one real-time PCR assay

The all-in-one real-time PCR assay was carried out in an Eppendorf tube for the simultaneous detection of *ompA* gene in the SFGR, segment L in both SFTSV and HTNV, and the house-keeping gene *ACTB* with a Gentier 96E/96R real-time thermocycler (Tianlong, Xi’an, China). We optimized the all-in-one real-time PCR assay by using SFGR-*ompA*, SFTSV-L, HTNV-L1 and HTNV-L2 as the PCR templates. We searched for the optimal temperature for PCR reaction between 58°C and 62.5°C. Subsequently, the results were analyzed using Medtl System software, and the optimal reaction temperature was determined based on the Ct values and fluorescence intensity of the reaction. Then, the primer concentration was optimized within the range of 200 to 600 pmol/mL, and the probe concentration was optimized within the range of 100 to 350 pmol/mL using the matrix method. The optimal primer-probe concentration was determined based on the Ct values of the reaction.

### 2.7 Evaluation of PCR efficiency, LOD and precision of the all-in-one real-time PCR assay

The amplification efficiency of the all-in-one real-time PCR assay was deduced from standard curves as described previously [29], which were generated by plotting the Ct values versus the log_10_ DNA concentration of the standards followed by constructing a linear regression equation. To construct the standard curve, six 10-fold dilutions of the plasmids SFGR-*ompA*, SFTSV-L, and HTNV-L2 starting with ~10^6^ copies/μL and ending with ~10^1^ copies/μL, were yielded, respectively.

LOD was determined according to Chinese National Standard GB/T 37871–2019. In brief, plasmids standards at 10^3^ and 10^1^ copies/μL were absolutely quantified using a digital PCR assay, then were serially two-fold diluted to be about 1 copies/μL (**S1–S3 Figs**). Each diluted plasmid was run in 20 replicates to determine the LOD of the all-in-one real-time PCR assay.

The precision of the all-in-one real-time PCR assay was evaluated according to EP15-A2 [30]. In brief, the evaluation was performed per day with three replicate samples at each of two concentrations (~10^4^ and ~10^0^ copies/μL) daily for five days. Imprecision was assessed by using the coefficient of variation (CV) on the Ct values.

### 2.8 Analysis of specificity and interference

A total of 4 serum samples from other bloodborne viruses were adopted to analyze specificity. Then they were separately tested using the all-in-one real-time PCR assay to investigate nonspecific amplification. In addition, single or multiple target nucleic acids at a concentration of LOD were performed on the all-in-one real-time PCR assay without or with these nucleic acids from other bloodborne viruses, then the statistical difference was calculated by an independent samples *t*-test on the Ct values to evaluate interference from HBV, HCV, EBV and CMV nucleic acids by SPSS.

### 2.9 Evaluation of the accuracy of the all-in-one real-time PCR assay

To evaluate of accuracy of the all-in-one real-time PCR assay, 321 serum or nucleic acid samples were tested. The results were compared to those reported previously. Sensitivity, specificity, positive and negative predictive values (PPV and NPV) were calculated. 37 was a Ct value measured from the concentration of LOD. Ct_*ACTB*_ were calculated from the Ct values of 225 characterized samples with mean and standard deviation.

## 3. Results

### 3.1 Optimal conditions for the all-in-one real-time PCR assay

**Fig 2:**
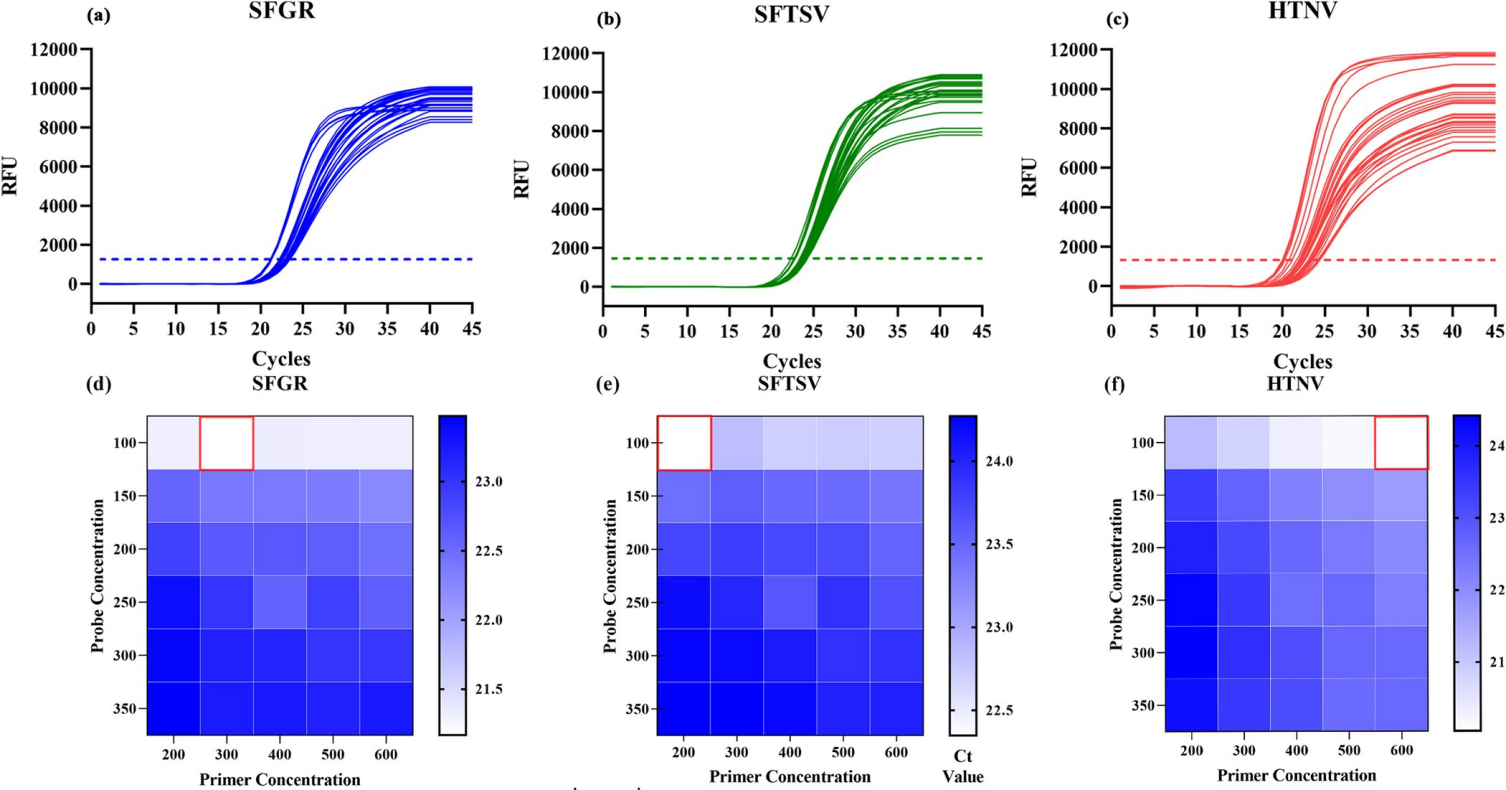
Primer and probe concentration optimization. (a) SFGR’s amplification curve; (b) SFTSV’s amplification curve; (c) HTNV’s amplification curve; (d) Best primer and probe concentration of SFGR; (e) Best primer and probe concentration of SFTSV; (f) Best primer and probe concentration of HTNV.

Our experimental data indicated that the optimal annealing/extension temperature was 60°C (**S2 Table**). As seen in **Fig 2**, the optimal concentration of primers ompA-F/R, DL-F/R, HL-F1/R1 and HL-F2/R2 is 300 pmol/mL, 200 pmol/mL, 600 pmol/mL and 600 pmol/mL, respectively. While the optimal concentration of all probes is 100 pmol/m. Finally, the volume of the all-in-one real-time PCR was 25 μL, comprising 5.0 μL of 5× Neoscript Fast RT Premix Buffer, 1.0 μL of 25× Neoscript Fast RTase/UNG Mix, 0.5 μL of an ompA-F/R mixture (300 pmol/mL), 0.5 μL of a DL-F/R mixture (200 pmol/mL), 0.5 μL of a HL-F1/R1 mixture (600 pmol/mL), 0.5 μL of a HL-F2/R2 mixture (600 pmol/mL), 0.5 μL of an ACTB-F/R mixture. 1 μL each of ompA-P, DL-P, HL-P1, HL-P2 and ACTB-P (100 pmol/mL), 2 μL of a template and 9.5 μL of ddH_2_O. The optimized thermal cycling conditions for amplification were as follows: 1 cycle of reverse transcription at 50°C for 15 min, and pre-denaturation at 95°C for 3 min, followed by 45 cycles of denaturation at 95°C for 10 s and annealing/extension at 60°C for 30 s. Monitoring of fluorescence occurred at the end of extension phase.

### 3.2 PCR efficiency, LOD and precision of all-in-one real-time PCR assay

**Fig 3:**
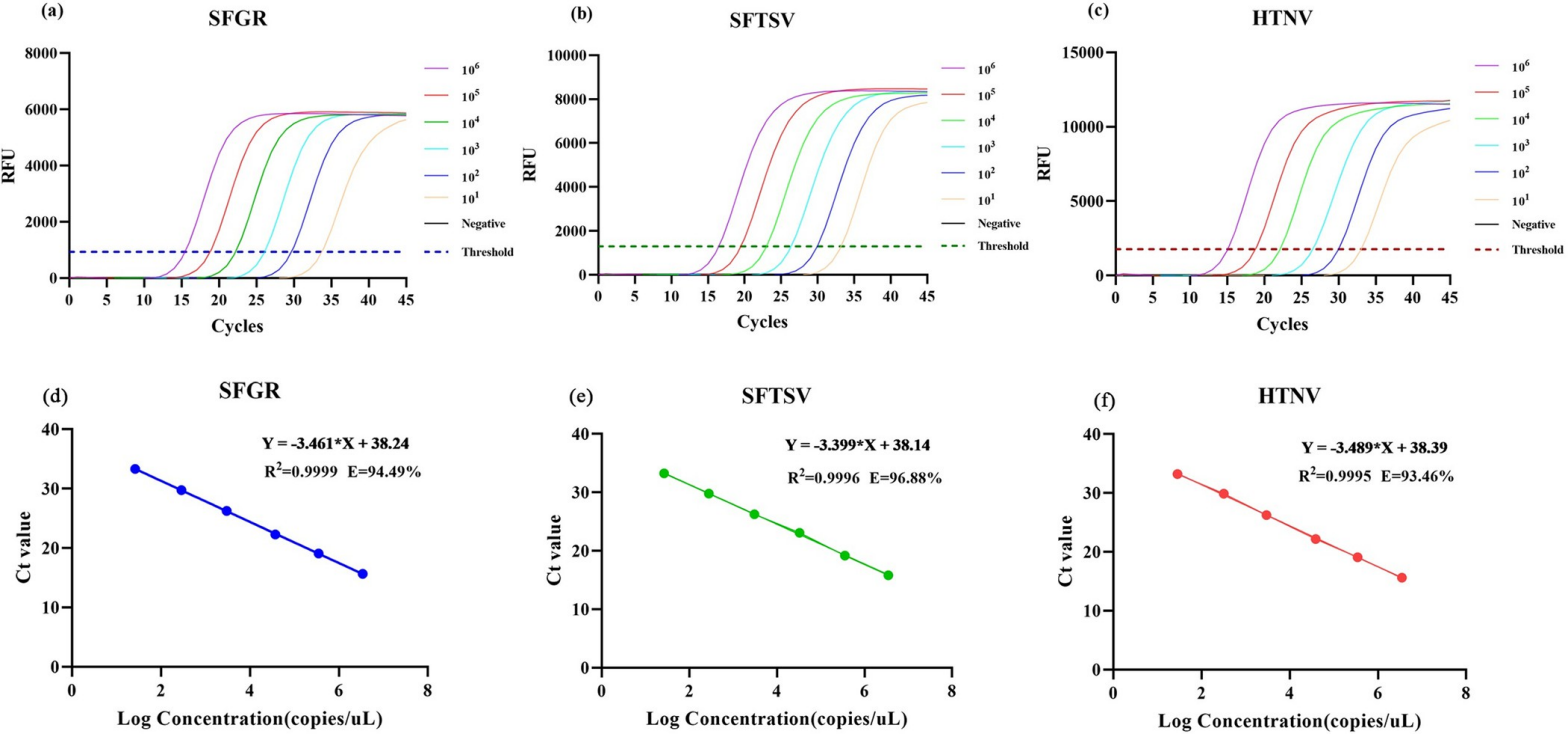
Amplification curves and standard curve construction used about 10^1^~10^6^ copies/μL plasmids. (a) SFGR’s amplification curve; (b) SFTSV’s amplification curve; (c) HTNV’s amplification curve; (d) SFGR’s standard curve; (e) SFTSV’s standard curve; (f) HTNV’s standard curve; The all-in-one real-time PCR assay reaction is performed using the optimal primer concentration. 95°C for 3 min; denaturation at 95°C for 10 s; annealing/elongation at 60°C for 30 s. 45 cycles.

Serial dilutions of three plasmids were co-amplified using the all-in-one real-time PCR assay to construct standard curves. Three linear standard curves were obtained within the range of 10^1^−10^6^ copies/μL with regression coefficients R^2^ ranging from 0.9995 to 0.9999, and amplification efficiencies ranging from 93.46% to 96.88% (**Fig 3**). The LOD were determined to be 1.108 copies/μL for the SFGR-*ompA*, 1.075 copies/μL for the SFTSV-L and 1.006 copies/μL for the HTNV-L with a detection rate of more than 95% (**S3 Table**). Regardless of target nucleic acids, the within-run CVs ranged from 0.53%~1.99%, whereas the within-laboratory CVs were limited to the range between 0.79% and 2.15% (**Table 2**).

**Table 2:** Within-run and within-laboratory reproducibility of the all-in-one real-time PCR assay.

Pathogen	Standard concentration (copies/μL)	Within-run			Within-laboratory		
		Mean Ct	SD	CV (%)	Mean Ct	SD	CV (%)
SFGR-*ompA*	32,629.9	20.46200	0.10752	0.53	20.61280	0.16201	0.79
	2.217	34.87633	0.35874	1.03	35.17633	0.63580	1.81
**SFTSV-L**	36,294.4	21.95167	0.20802	0.95	21.83000	0.30018	1.38
	2.151	35.80333	0.55486	1.55	35.45907	0.68253	1.92
**HTNV-L**	35,303.3	20.41267	0.14856	0.73	20.99873	0.38912	1.85
	2.013	34.00400	0.67554	1.99	34.41060	0.74143	2.15

SFGR: spotted fever group rickettsiae; SFTSV: severe fever with thrombocytopenia syndrome virus; HTNV: hantaan virus.

### 3.3 Specificity and the anti-interference ability of the all-in-one real-time PCR assay

**Fig 4:**
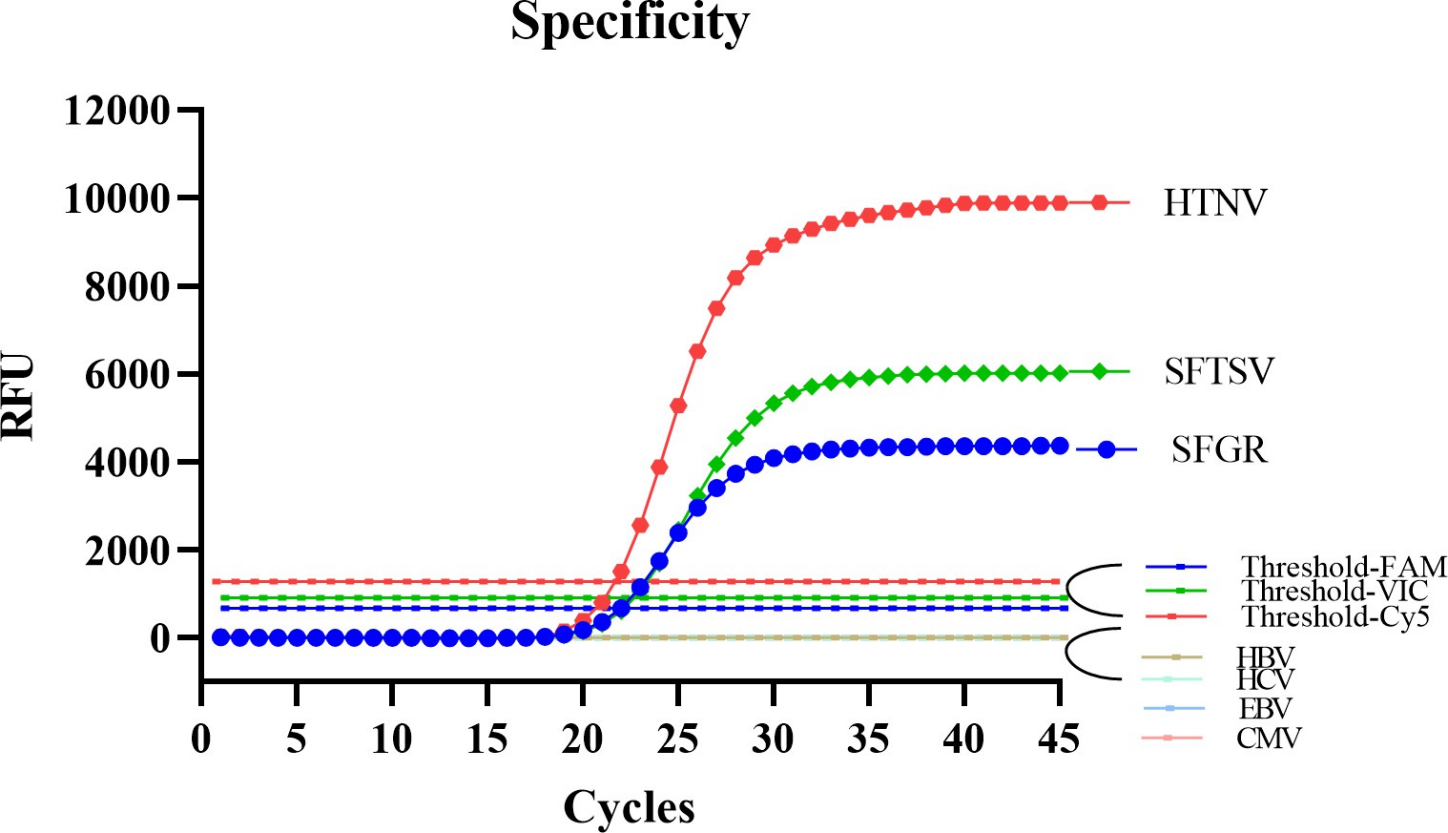
Results of specificity experiment. Only the positive control well has an amplification curve; HBV, HCV, EBV and CMV show no reaction curves.

To assess specificity of the all-in-one real-time PCR assay, the nucleic acids of four serum samples which were previously tested positive for HBV-DNA, HCV-RNA, EBV-DNA and CMV-DNA, respectively, were used as targets. All of them did not produce positive signals and only plasmids SFGR-*ompA*, SFTSV-L, and HTNV-L yielded classical “S” type curves, indicating the detection method’s high specificity (**Fig 4**). Then the plasmids were tested to evaluate the interference from HBV-DNA, HCV-RNA, EBV-DNA and CMV-DNA. The resulting Ct values are listed in **Table 3**. It was found there was no statistical difference in the Ct values between single template and multiple template for all three targets (P_SFGR_ = 0.186, P_SFTSV_ = 0.612, P_HTNV_ = 0.298). It was also shown that the Ct value fluctuation was less than 1.1 when the non-target bloodborne virus nucleic acids were added (**Table 3**).

**Table 3:** The Ct values variation after adding non-specific nucleic acids.

pathogens		Ct values						
					HBV	HCV	EBV	CMV
**Single template**	SFGR-*ompA*	34.996	35.262	35.066	34.965	35.707	35.207	35.574
	**SFTSV-L**	36.926	36.645	36.520	35.918	36.121	36.121	35.887
	**HTNV-L**	35.621	35.793	35.652	36.355	36.059	36.309	35.941
Multiple template^a^	SFGR-*ompA*	35.316	35.496	35.137	35.723	35.699	36.160	35.988
	**SFTSV-L**	36.707	36.707	36.426	35.730	35.527	36.387	35.910
	**HTNV-L**	35.301	35.598	35.699	36.027	36.293	35.590	36.395

a: SFGR, SFTSV and HTNV were mixed as a template (SFGR: 1.108 copies/μL; SFTSV: 1.075 copies/μL; HTNV: 1.006 copies/μL).

### 3.4 Evaluation accuracy of all-in-one real-time PCR assay with samples

Judgment criteria are as follows: (a) Positive: Ct<37 and a typical amplification curve is observed (Ct_*ACTB*_ 22.48–27.67). (b) Negative: no Ct value and no amplification curve (Ct_*ACTB*_ 22.48–27.67). (c) Retesting: Ct>37 and a typical amplification curve is observed. If the retest result is the same as mentioned above, it is considered positive; otherwise, it is deemed negative (Ct_*ACTB*_ 22.48–27.67). (d) Unqualified DNA sample: Ct_*ACTB*_ < 22.48 and Ct_*ACTB*_ > 27.67.

A total of 321 samples were collected to evaluate the accuracy of the all-in-one real-time PCR assay. It was found the sensitivity, specificity, positive and negative predictive value for testing SFGR-*ompA* and SFTSV-L nucleic acids were all 100%, whereas the detection of HTNV-L was 97%, 100%, 100% and 99.6%, respectively (**Table 4**).

**Table 4:** The sensitivity, specificity, PPV and NPV of the all-in-one real-time PCR assay.

Pathogen	Outcome	Control method	All-in-one real-time PCR assay	Sensitivity	Specificity	PPV^a^	NPV^b^
SFGR-*ompA*	Positive	17	17	100%	100%	100%	100%
	Negative	225	225				
**SFTSV-L**	Positive	46	46	100%	100%	100%	100%
	Negative	225	225				
**HTNV-L**	Positive	33	32	97%	100%	100%	99.6%
	Negative	225	225				

a: Positive Predictive Value; b: Negative Predictive Value.

## Discussion

In the present study, the all-in-one real-time PCR was successfully established to simultaneously detect the nucleic acids from SFGR, SFTSV and HTNV. The SFGR-*ompA*, as well as the segment L of SFTSV and HTNV, were used as targets for amplification. It was worth noting that two sets of primers and probes were used to amplify segment L of HTNV due to high variation in China [31]. China had the highest number of HFRS cases worldwide. There exist numerous branches of HTNV, with highly diverse genetics in Heilongjiang, Shanxi, Liaoning, Shandong, Jilin, Hebei, Hunan, Zhejiang, Jiangxi, Jiangsu and Hubei provinces [32]. Recently, the genetic evolution analysis of the L segment revealed that the viral sequences prevalent in Hubei province cluster together, forming a distinct lineage with genetic variations from viruses in other regions [18]. To enhance our detection capabilities, we have designed two sets of primers and probes for HTNV infection. HTNV-L2 is primarily utilized to detect the unique lineage (HV004-like) that is prevalent in Hubei province, whereas HTNV-L1 is employed for detecting infection in other regions.

The all-in-one real-time PCR method demonstrated high sensitivity, with the ability to detect approximately 1 copies/μL of the virus genome. It also exhibited an excellent linear range between 10^6^ and 10^1^ copies/μL, where the regression coefficients R^2^ for the target nucleic acids ranged from 0.9995 to 0.9999 and the PCR amplification efficiencies ranged from 93.46% to 96.88%, with a dynamic range of six orders of magnitude (10^1^−10^6^ copies/μL). To assess its specificity, we confirmed that other related viruses such as HBV, HCV, EBV and CMV did not produce positive signals and did not affect the Ct value of the positive target, indicating the high specificity of the detection method. Furthermore, the detection method showed high reproducibility, with relatively small variations observed in both within-run and within-laboratory capabilities. The average coefficients of variation (CVs) for within-run and within-laboratory were limited to the range between 0.53%-2.15%, which is considered acceptable in terms of reproducibility. Using the all-in-one real-time PCR method, we successfully detected SFGR, SFTSV and HTNV in 321 expected samples. Among these samples, 17 were identified as SFGR positive, 46 as SFTSV positive and 33 as HTNV positive. The detection system’s sensitivity, specificity, PPV and NPV for SFGR and SFTSV were all 100%, while the detection of HTNV was 97%, 100%, 100% and 99.6%. These results demonstrate the effectiveness of the all-in-one real-time PCR assay in detecting and differentiating these pathogens in clinical samples.

Since zoonotic diseases like SFTSV, HTNV and SFGR are often prevalent in economically underdeveloped areas, the risk of misdiagnosis is high due to their similar clinical symptoms. Therefore, it is crucial to develop and evaluate a multiplex assay that can detect and identify these similar pathogens simultaneously. So far, there only exists single real-time fluorescence PCR detection method for SFTSV and HTNV. In comparison, our all-in-one real-time PCR method can simultaneously detect SFGR, SFTSV, and HTNV, making it advantageous for identifying and detecting similar symptoms in patients, as well as conducting large-scale screenings in epidemic areas [33–35]. In comparison to the immunochromatographic assay (ICA), which is a cheaper and more convenient on-site testing method but has limitations in terms of sensitivity and delayed detection windows, our method offers a longer detection window and higher sensitivity [36]. Compared to the SFTSV CRISPR detection method established by Zou et al., our detection method offers lower detection limits (about 1 copy/μL), reduced costs, and the ability to conduct multiple detections. However, it should be noted that our method has a slightly longer detection time of 60 min when compared to their CRISPR detection method [37,38]. For other methods, such as virus isolation, a long experimental cycle of about 10 to 15 days is required in comparison with PCR, thus rendering them unsuitable for clinical promotion. Metagenomic sequencing offers the advantage of detecting a wide range of pathogens without bias and conducting systematic geographical analysis, but its lack of standardization, limited personnel expertise, and high cost hinder its widespread application in clinical practice [39]. Overall, the all-in-one real-time PCR assay we have established has the advantages of lower detection limits, lower costs, shorter processing time, and longer detection window period, making it suitable for application in primary medical institutions and remote areas.

Our research demonstrates that the all-in-one real-time PCR assay testing has high sensitivity, specificity and reproducibility. Additionally, the turnaround time for experiments is approximately 1.5 hours, including nucleic acid extraction steps. This makes it a high-throughput, reliable and cost-effective diagnostic and screening tool for early clinical diagnosis of acute-phase SFTSV, HTNV and SFGR. Consequently, the all-in-one real-time PCR assay enables the simultaneous detection of multiple pathogens in a single reaction system, offering great potential for future clinical point-of-care applications. This advancement holds promise in assisting with early and accurate diagnosis, as well as contributing to effective public health management and infectious disease control.

## Supporting information

S1 Table The conserved region sequences used for constructing the plasmids. (DOCX)

S2 Table The Ct value of temperature optimization for SFGR, SFTSV and HTNV. (DOCX)

S3 Table Detection rate of LOD concentration gradient. (DOCX)

S1 Fig Digital PCR results of SFGR 10^3^ and 10^1^ copies/μL concentration. (DOCX)

S2 Fig Digital PCR results of SFTSV 10^3^ and 10^1^ copies/μL concentration. (DOCX)

S3 Fig Digital PCR results of HTNV 10^3^ and 10^1^ copies/μL concentration. (DOCX)

S1 Data Separate tables documents containing basic numerical data, statistical analysis and original pictures for Figs 1, 2, 3 and Tables 2, 4 of this study. (XLSX)
