# Supplementary material for: Development of an all-in-one real-time PCR assay for simultaneous detection of spotted fever group rickettsiae, severe fever with thrombocytopenia syndrome virus and hantaan virus prevalent in central China
Source: PLoS Negl Trop Dis. 2024 Jul 16;18(7):e0012024. doi: 10.1371/journal.pntd.0012024 (PMC11280241; doi:10.1371/journal.pntd.0012024)
Supplement: S1 Table — (DOCX) [file pntd.0012024.s001.docx]

**S1 Table. The conserved regions sequences used for constructing the plasmids.**

| **Pathogen** | **Conserved regions sequences（5’-3’）** |
| --- | --- |
| **SFGR-*ompA*** | 5’-CCAGATACAAAATATACGGTAATATCTGCAGAAACAGCAGGTGATTTAAAGCCTACTCCTAAAGAGAATGTTAAAATAACTATTAATAATGACAACCGTTTTGTTGATTTTACTTTTGATGCATCGACTTTAACGTTATTTGCAGAGGATATAGCTGCAGATGTTATAGATGAAGATTTTGCACCGGGTGGACCGCTTGTAAGTATCCCAAATGCTGCAAATATAAAGAAATCGCTTGAGTTAATGGAGGATGCTCCCAATGGTTCAGATGCACGTCAAGCTTTCAATAACTTTGGTCTAATGACACCGCTACAGGAAGCAGATGCTACAACTCATCTCATTCAAGATGTTGTAAAACCTAGCGATACTATAGCTGCCGTTAATAATCAAGTTGTAGCAAGTAATATATCAAGTAATATAACTGCTCTAAATGCTAGAATGGATAAAGTACAAGTAGCGAATAAAGGTCCTGTTTCTTCCGGTGATGAAGATATGGATGCTAAGTTTGGTGCGTGGATAAGCCC-3’ |
| **SFTSV-L** | 5’-ACACAGAGACGCCCAGATGAACTTGGAAGTGCTTTGTGGTAGGATAAACGTGGAGAATGGGCTGTCTCTTGGAGAACCAGGCCTGTACGACCAAATCTACGATAGGCCAGGGCTTCCAGACCTAGATGTGACTGTCGATGCCACTGGTGTTACAGTGGACATAGGGGCTGTGCCAGACTCAGCATCACAATTGGGCTCATCAATCAACGCTGGGTTGATCACAATCCAGCTCTCAGAGGCATATAAGATCAATCATGACTTCACGTTTTCTGGCCTGTCAAAGACAACAGACCGACGCCTCTCAGAGGTATTCCCCATTACCCATGATGGTTCTGATGGGATGACCCCTGATGTGATTCACACAAGATTGGATGGAACCATTGTGGTGGTTGAATTTTCAACCACTAGGAGCCATAACATTGGGGGCCTGGAGGCAGCATATAGGACAAAGATAGAAAAATATAGGGACCCAATCTCAAGACGTGTTGATATCATGGAGA-3’ |
| **HTNV-L1** | 5’-TGAAAGTTCATACACAGGAGACCTATTAATCAGCACAGAGGAAACTGAATCAAAGAAAATGAGGGGCATAGTGAAAATACTTGAGCCTGTTAGATTGATTAAAAGCTGGGTATCACGTGGGTTATCTATTGAGAAAGTATATAGTCCTGTTAATATTATCTTAATGTCACGGTATATCTCCAAAACATTTAATTTGAGTACCAAACAGGTCTCATTATTAGATCCATATGATTTAACAGAATTAGAGAGCATTGTCCGAGGATGGGGAGAATGTGTTATTGACCAGTTCGAAAGTCTCGATAGAGAGGCTCAGAATATGGTTGTTAATAAAGGAATATGCCCTGAGGATGTTATTCCTGATTCATTATTTTCATTTAGGCACACCATGGTACTGTTGAGGAGGTTATTCCCGCAGGATTCTATTTCCTCTTTCTATTAGGCTTTCTTTCTTTTTCATTTTCCGGAGCATACTACTACTA-3’ |
|  |  |
| **HTNV-L2** | 5’-GTCTTTTCAAGTTCTTAATGAATTTATCTGAAGAAACATTTCAGCATGAGAGGTTAGGGCAGTTTTCATTTATCGGGAAGGTGCAGTGGAAAATTTTTACACCTAAATCTGAATTTGAGTTTGCTGATATGTACACATCTAAATTTCTAGAGCTCTGGAGTAATCAGCACATAACATACGATTACATCATTCCCAAAGGTAGGGATAATTTGCTTATTTACTTAGTTAGGAAGCTAAATGACCCGAGCATTGTAACTGCCATGACCATGCAATCACCTTTACAATTACGATTCCGTATGCAAGCAAAGCAACATATGAAAGTATGCAGGCTTGACGGGGAGTGGGTTACTTTCAGAGAAGTTTTAGCTGCAGCAAATAGCTTTGCTGAAAGTTACAGTCCAACTAGTCAGGATATAGACTTATTTCAAACATTAACAAGTTGCACATTTTCTAAAGAATATGCATGGAAAGATTTTTTAAATGGAATTCATTGTGATGTTATCCCGACAAAACAAGTTCAGAGGG-3’ |
