# Supplementary material for: Development of an all-in-one real-time PCR assay for simultaneous detection of spotted fever group rickettsiae, severe fever with thrombocytopenia syndrome virus and hantaan virus prevalent in central China
Source: PLoS Negl Trop Dis. 2024 Jul 16;18(7):e0012024. doi: 10.1371/journal.pntd.0012024 (PMC11280241; doi:10.1371/journal.pntd.0012024)
Supplement: S2 Table — (DOCX) [file pntd.0012024.s002.docx]

S2 Table. The Ct value of tempreture optimization for SFGR, SFTSV and HTNV.

|  | 58℃ | 58.4℃ | 59.3℃ | 60℃ | 61.6℃ | 62.5℃ |
| --- | --- | --- | --- | --- | --- | --- |
| SFGR | 26.160 | 26.027 | 26.020 | 26.012 | 26.098 | 26.480 |
| SFTSV | 27.590 | 27.520 | 27.535 | 27.512 | 27.574 | 27.684 |
| HTNV | 24.082 | 23.965 | 23.738 | 23.918 | 24.395 | 25.176 |
