# Supplementary material for: Development of an all-in-one real-time PCR assay for simultaneous detection of spotted fever group rickettsiae, severe fever with thrombocytopenia syndrome virus and hantaan virus prevalent in central China
Source: PLoS Negl Trop Dis. 2024 Jul 16;18(7):e0012024. doi: 10.1371/journal.pntd.0012024 (PMC11280241; doi:10.1371/journal.pntd.0012024)
Supplement: S3 Table — (DOCX) [file pntd.0012024.s003.docx]

**S3 Table. Detection rate of LOD concentration gradient.**

| **Number** | **Ct value** | | |
| --- | --- | --- | --- |
|  | **SFGR** | **SFTSV** | **HTNV** |
| 1 | 36.449 | 34.902 | 35.441 |
| 2 | 35.980 | 35.316 | 35.770 |
| 3 | 36.699 | 35.863 | 35.004 |
| 4 | 35.645 | 36.129 | 35.223 |
| 5 | 36.145 | 35.371 | 35.676 |
| 6 | 36.332 | 35.098 | 36.004 |
| 7 | 35.934 | 36.551 | 35.027 |
| 8 | 36.465 | 35.863 | 35.551 |
| 9 | 35.621 | 36.293 | 35.551 |
| 10 | 35.270 | 36.691 | 36.348 |
| 11 | 36.574 | 36.785 | 35.184 |
| 12 | 36.832 | 35.512 | 35.340 |
| 13 | 36.660 | 35.934 | 35.465 |
| 14 | 35.160 | 35.441 | 35.082 |
| 15 | 37.012 | 34.809 | 35.340 |
| 16 | 37.473 | 35.457 | 35.379 |
| 17 | 36.559 | 35.426 | 35.840 |
| 18 | 36.402 | 37.160 | 35.754 |
| 19 | 36.676 | 35.863 | 35.613 |
| 20 | 35.840 | 35.941 | 35.699 |
