# Supplementary material for: Development of an all-in-one real-time PCR assay for simultaneous detection of spotted fever group rickettsiae, severe fever with thrombocytopenia syndrome virus and hantaan virus prevalent in central China
Source: PLoS Negl Trop Dis. 2024 Jul 16;18(7):e0012024. doi: 10.1371/journal.pntd.0012024 (PMC11280241; doi:10.1371/journal.pntd.0012024)
Supplement: S3 Fig — (DOCX) [file pntd.0012024.s006.docx]

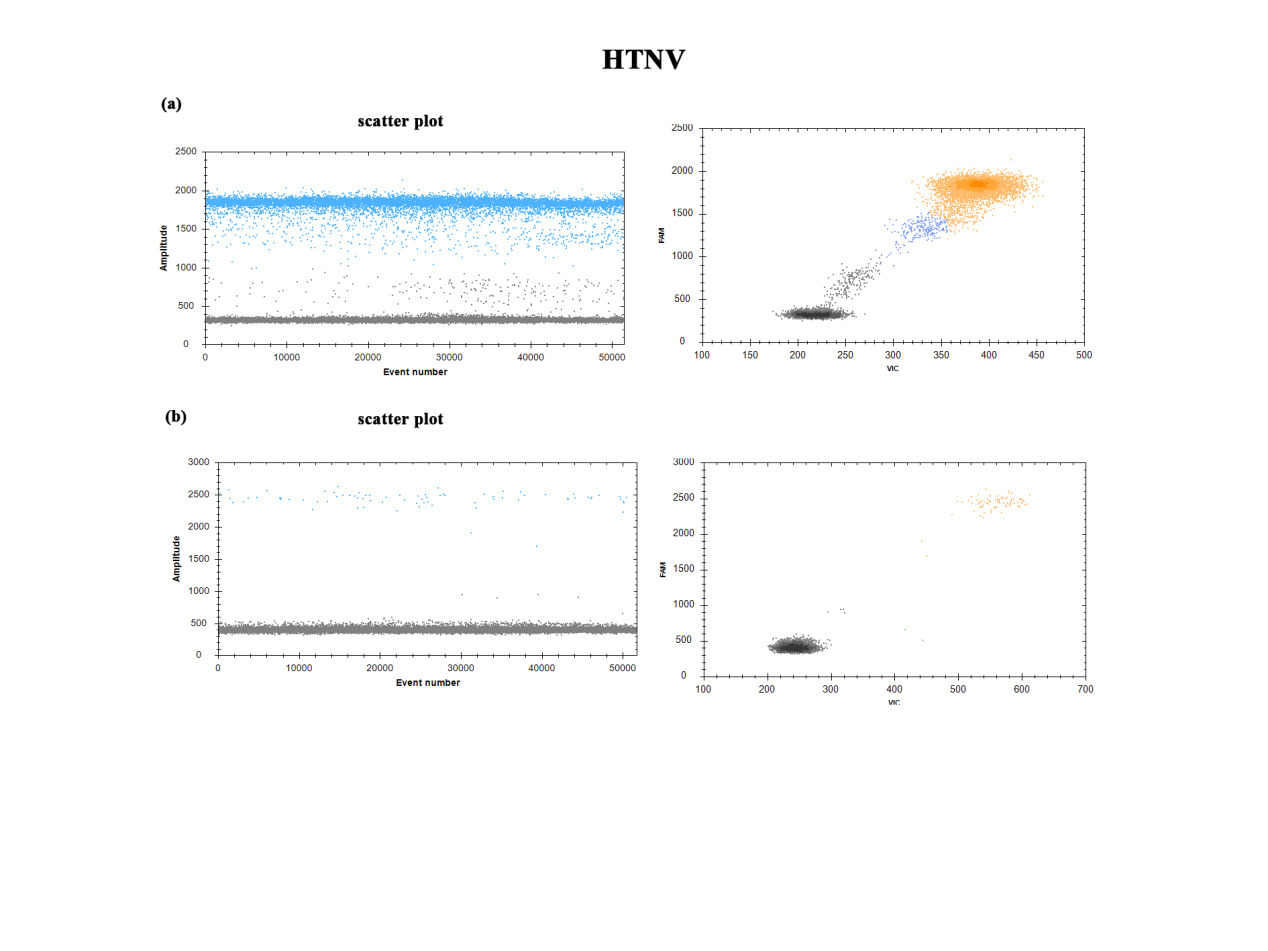


S3 Fig. Digital PCR results of HTNV 10^3^ and 10^1^ copies/μL concentration. (a) The scatter plot of HTNV 10^3^ copies/μL about 3,530.33 copies/μL; (b) The scatter plot of HTNV 10^1^ copies/μL about 20.13 copies/μL.
